# Attention Alters Population Spatial Frequency Tuning

**DOI:** 10.1523/JNEUROSCI.0251-25.2025

**Published:** 2025-05-22

**Authors:** Luis D. Ramirez, Feiyi Wang, Sam Ling

**Affiliations:** ^1^Department of Psychological & Brain Sciences, Boston University, Boston, Massachusetts 02215; ^2^Center for Systems Neuroscience, Boston University, Boston, Massachusetts 02215

**Keywords:** attention, fMRI, population tuning, spatial frequency

## Abstract

Spatial frequency (SF) selectivity serves as a fundamental building block within the visual system, determining what we can and cannot see. Attention is theorized to augment the visibility of items in our environment by changing how we process SFs. However, the specific neural mechanisms underlying this effect remain unclear, particularly in humans. Here, we used functional magnetic resonance imaging to measure voxel-wise population SF tuning (pSFT), which allowed us to examine how attention alters the SF response profiles of neural populations in the early visual cortex (V1–V3). In the scanner, participants (five female, three male) were cued to covertly attend to one of two spatially competing letter streams, each defined by low or high SF content. This task promoted feature-based attention directed to a particular SF, as well as the suppression of the irrelevant stream's SF. Concurrently, we measured pSFT in a task-irrelevant hemifield to examine how the known spatial spread of feature-based attention influenced the SF tuning properties of neurons sampled within a voxel. We discovered that attention elicited attractive shifts in SF preference, toward the attended SF. This suggests that attention can profoundly influence populations of SF preference across the visual field, depending on task goals and native neural preferences.

## Significance Statement

The spatial frequency (SF) preference of neural populations in the early visual cortex governs the coarse and fine details we can see. However, the brain is limited in what it can process, requiring selective attention to prioritize relevant over irrelevant details. Although SF is fundamental to visual processing, it remains unclear how selective attention to SF alters population-level responses to SF. Using fMRI, we measured SF preferences in V1–V3 while participants deployed feature-based attention to one of two competing stimuli solely defined by their SF. We found that attention produced attractive shifts in preferences across the visual field, toward the attended SF, demonstrating that voluntary attention can flexibly reshape SF preferences in the early visual cortex.

## Introduction

Signals in our brain are constantly vying for metabolic resources (Lennie, 2003), imposing a limit on the content and fidelity of information available for processing from moment to moment (Simoncelli and Olshausen, 2001; Carandini et al., 2005). Attention is theorized to play a key role in selectively regulating competing representations, prioritizing the processing of behaviorally relevant features, while suppressing the irrelevant (Lee et al., 1999; Carrasco, 2011; Maunsell, 2015; Wu, 2024). Indeed, attention has been known to boost the gain of populations that represent attended items (Maunsell and Treue, 2006; Carrasco, 2011; Ling et al., 2015; Maunsell, 2015; Liu, 2019), an effect that has been reported neurally in animals (Treue and Maunsell, 1996; Treue and Martínez-Trujillo, 1999; Martinez-Trujillo and Treue, 2004; David et al., 2008; Zhang and Luck, 2009; Cohen and Maunsell, 2011) and humans (Sasaki et al., 2001; Serences et al., 2009; Pestilli et al., 2011; Klein et al., 2014; Foster and Ling, 2022), as well as psychophysically (Ling et al., 2009; Herrmann et al., 2012; Fang and Liu, 2019). Moreover, these modulatory effects of attention are believed to impinge upon a cornerstone selective property in early vision: spatial frequency (SF) processing (Carrasco et al., 2006; David et al., 2008). The spatial frequencies a neural population selectively responds to are synonymous with the density of spatial detail it can encode (Blakemore and Campbell, 1969; Braddick, 1981; De Valois et al., 1982). Therefore, given the critical role of this selectivity in governing what we can and cannot see at any given moment, the ability to augment it would grant attention significant power to shape perception (Anton-Erxleben and Carrasco, 2013). However, while SF is implicated as a modulatory target for resolving competition between representations (Sowden and Schyns, 2006; Anton-Erxleben and Carrasco, 2013), there is a gap in our understanding of how attention modulates population responses to SF to resolve competition, particularly in human cortex (Pouget et al., 2000; Jazayeri and Movshon, 2006; Sowden and Schyns, 2006; Fang and Liu, 2019).

To bridge this gap, we leveraged a model-based fMRI technique, population spatial frequency tuning (pSFT), which can efficiently estimate both the preferred SF (pSFT peak) and the range of SFs that elicit a response (pSFT bandwidth) in neural subpopulations sampled within a voxel (Aghajari et al., 2020). We paired this technique with a novel feature-based attention paradigm, in which participants selectively attended one of two streams of letters, defined by their distinct SF properties. In doing so, we were able to assess voxel-wise changes in SF processing within and across early visual cortices (V1–V3). Our results revealed profound shifts in the peak SF preference and bandwidth of subpopulations throughout the early visual cortex that depended on the nature of the attentional task and stimuli. Specifically, we discovered that attention elicited substantial “attractive shifts” in SF preference toward the attended SF: subpopulations that innately preferred SFs lower than the attended item shifted higher and those that innately preferred higher SFs shifted lower. Evidently, feature-based attention has the power to flexibly alter SF tuning for individual subpopulations, shifting cortical SF preferences to dynamically process the qualities of attended items.

## Materials and Methods

### Subjects

Eight healthy adult volunteers (five female) between ages 22 and 33 (age, 27.3 ± 1.6; mean ±SEM) participated in the experiment. All subjects had normal or corrected-to-normal vision. This sample size was chosen to mirror the original population spatial frequency tuning mapping study by Aghajari et al. (2020). All subjects involved provided written consent and were reimbursed for their time. The Boston University Institutional Review Board approved the study.

### Apparatus and stimuli

In the testing room used for initial calibration and training, stimuli were displayed on a gamma-corrected Display++ LCD monitor (Cambridge Research; resolution, 1,440 × 1,080 pixels; refresh rate, 100 Hz; viewing distance, ∼114 cm, the distance needed to mimic the pixels per degree in the scanner setup), with no additional light sources in the room. Participants were seated with their chin on a padded chin rest and their forehead rested.

In the MRI scanner bore, stimuli were displayed on a linearized, gamma-corrected rear-projected screen (VPixx PROPixx DLP LED; resolution, 1,024 × 768 pixels; refresh rate, 60 Hz; viewing distance, ∼99 cm), with no additional light sources in the room. All stimuli were presented on a uniform gray background (mean luminance, ∼150 cd/m^2^). A dot was presented at the center of the display for fixation (diameter, 0.15° of visual angle). Stimuli were generated using MATLAB 2017b (The MathWorks Inc., 2017) and the Psychophysics Toolbox (Brainard, 1997) rendered on Ubuntu 18.04.3 LTS.

The visual display was partitioned into attended (task-relevant) and unattended (task-irrelevant) hemifields during task blocks. At the attended hemifield, two rapidly updating letter streams were superimposed (4 Hz character refresh rate; eccentricity, 3.5° horizontal from fixation; diameter, 3°). Each letter stream contained spatial frequency bandpass-filtered Sloan letters, with a center SF of 0.5 and 2 cpd, respectively (filter width, 0.2; Gaussian smoothing kernel width, 3). Letter characters consisted of A, C, D, J, K, L, M, P, S, V, X, Y, and Z. Letter characters did not repeat sequentially within a letter stream nor were the same letter presented simultaneously between letter streams. Target letters J and K had a 20% probability of occurrence. To avoid the effects of attentional blink (Dux and Marois, 2009), there was at least 500 ms between target letters within a letter stream.

At the unattended visual hemifield, a pseudorandom sequence of 40 SF-bandlimited noise stimuli was presented (10 Hz noise sample refresh rate), with center SFs that were logarithmically spaced from 0.1 to 12 cpd (filter width, 0.2). These stimuli were presented at 100% Michelson contrast and through a wedge aperture that subtended 5° from fixation (outer radius, 9.18°; inner radius, 0.32°). At center fixation, the dot changed from white to black between rest periods and task blocks. During task blocks, the dot would pseudorandomly change in luminance from 0 to 30, with the same target probability as the target letters. Stimulus presentation statistics were identical in every block.

Five subjects (S1, 2, 3, 4, and 7) had the probe stimuli on the left visual hemifield (pSFT analyzed from the right hemisphere), while the remaining subjects (S5, 6, and 8) had the probe stimuli on the right visual hemifield (pSFT analyzed from the left hemisphere). Results did not significantly differ when data were grouped and compared by probe hemisphere (Wilcoxon ranked sum test *p* > 0.05).

### Spatial frequency-bandpass filtering

All stimuli were SF-bandpass filtered in MATLAB 2017b scripts. The Sloan letters used (Pelli et al., 1988) were imported as TIFF files and resized so that the diameter of the letters spanned 3°. To resize the images appropriately, we calculated a pixel-based ratio between the letter size and image size to determine how much the image width needed to be resized to achieve the desired letter diameter. Next, the complement of each letter image was taken, reversing the luminance of each pixel so that the background changed from white to black and the letters from black to white. The *fft2* MATLAB function was used to apply a 2D fast Fourier transform (FFT) on the image, followed by *fftshift* to shift the DC component to the center of the frequency domain.

A 2D bandpass filter (σ=0.2) was generated with the *Bandpass2* function from *Psychtoolbox*. The filter matched the size of the image, and the radial cutoff frequencies, fLow and fHigh, were divided by the Nyquist frequency, $f_{\mathrm{Nyquist}}=1/(2\;*\;\mathrm{pixel}\;\mathrm{size}\;(\mathrm{cm}))$ to create a circularly symmetric filter. The 2D bandpass filter was smoothened with the *imgaussfilt* function with a standard deviation of 3 pixels for the letter images and a tenth of the pixels per degree of visual angle for the noise stimuli. The filter was normalized by subtracting the minimum value from each element and then dividing the difference by the range. Next, the normalized bandpass filter was applied to the shifted FFT image. To inverse the FFT and return the image to the spatial domain, the *ifftshift* and *ifft2* functions were applied to the image, in that order.

To finalize the images, the minimum value was subtracted from each element, and then the difference was divided by the range in the case of letter stimuli or by the maximum in the case of the noise stimuli. Finally, the filtered images were rectified (taking the absolute value) and converted to a visible range of pixel values between 0 and 255 for drawing by scaling every element by 127 and then adding 127.

### Eye tracking

An MRI-compatible EyeLink 1000 Plus infrared eye tracker (SR Research) was used to monitor gaze position and pupil size throughout the experiment at a sampling rate of 500 Hz. Each scan began with eye calibration and validation. Subjects were instructed to maintain fixation throughout the study. Eye data were analyzed with custom MATLAB scripts. Average pupil size, horizontal gaze position, and fixation stability [quantified as the bivariate contour ellipse area (Crossland et al., 2004)] were compared with a one-way ANOVA to confirm no significant differences between conditions at the group level.

### Main task

Each task run consisted of an initial 10 s blank period and six 40.5 s task blocks, each followed by a 10 s blank period (313 TRs per run). Before a task block, participants were briefly presented one of three color cues at fixation (1 s duration), indicating which task to perform: a low SF letter detection task at 0.5 cpd (“Attend LSF” condition), a high SF letter detection task at 2 cpd (“Attend HSF” condition), or a luminance change detection task at fixation (“Attend Fixation” condition). The task block began 30 ms after the cue offset. The fixation dot was black during task blocks and white during blank periods. Color cues were randomly assigned per subject, and task conditions were randomly interleaved, with 2 blocks per condition in each task scan (nine task scans per subject; 18 blocks per condition per subject).

In the “Attend Fixation” condition, subjects reported with a button press the detection of a brief (250 ms) change in luminance, from black to gray (0–30), at central fixation. In the letter detection task (“Attend LSF” and “Attend HSF” conditions), participants were cued to covertly attend to one of two superimposed letter streams and report the detection of a target letter J or K by pressing the left or right button, respectively (response window, 1 s).

Before scanning, participants completed a 1 h stimulus calibration and training session. Calibration involved participants maintaining fixation while adjusting the alpha level of the LSF letter stream, which began at an alpha level of 127 by default (where 0 means complete transparency and 255 means complete opacity). Participants could adjust the size of the increments if needed. Participants were instructed to reach an alpha level that allowed for comparable detection of the target letters between letter streams, with emphasis made on achieving an alpha level where neither letter stream appeared to dominate the other.

When participants found a satisfactory alpha level for the LSF letter stream, the new alpha level was applied to training. The training involved performing at least three runs of the main task (at least six blocks per condition in total). If the difference in performance between conditions was >10%, participants either completed additional training runs or could readjust the alpha level of the LSF letter stream. Either case was followed by additional training until the difference in performance between “Attend LSF” and “Attend HSF” conditions was roughly 5–10% or time constraints were met.

Outside the scanner bore and on scan day, subjects practiced the task on a Lenovo ThinkPad laptop to reconfirm that performance was comparable (three runs maximum so that we had enough time to complete all nine scan runs of the main task). In the scanner bore and before the main task, subjects had an opportunity to adjust the LSF letter stream alpha level (mean alpha level = 168, SD = 31.3). After confirming the alpha level, participants completed anatomical scans, two probe localizer scans, and nine main task scans. The localizer scans were not used in the final analyses due to the letter stream spatial localizer being kept at 2.5° eccentricity (the settings from a pilot study) instead of the 3.5° eccentricity used in the main experiment. Behavioral performance in the main experiment was measured as percent correct and analyzed with a one-way ANOVA to confirm no significant differences in performance between conditions at the group level.

### Functional magnetic resonance imaging data acquisition

All high-resolution brain data were collected at the Boston University Cognitive Neuroimaging Center, which houses a Siemens 3 T Prisma scanner equipped with a 64-channel head coil provided by Siemens Healthcare. A whole-brain anatomical scan was acquired with a T1-weighted multiecho magnetization-prepared rapid acquisition gradient echo (MPRAGE) sequence (1.2 mm^3^; FOV, 192 mm × 192 mm × 176 mm; fractional anisotropy flip angle (FA), 7°; TR, 2,200 ms; TE, 1.57 ms; TI, 1,100 ms; Van Der Kouwe et al., 2008). All BOLD data for the main task were acquired with a T2*-weighted in-plane echo planar imaging (EPI) pulse sequence with simultaneous multislice (SMS) imaging and a field of view perpendicular to the calcarine sulcus [2 mm^3^ voxels; FOV, 936 mm × 936 mm × 313 mm (probe localizer FOV, 936 mm × 936 mm × 320 mm); FA, 64°; TR, 1,000 ms; TE, 30 ms; Moeller et al., 2010; Xu et al., 2013). BOLD data for the population receptive field (pRF) mapping session were acquired with a T2*-weighted in-plane EPI-SMS imaging sequence and a FOV perpendicular to the calcarine sulcus, but with the following parameters: 2 mm^3^ voxels; FOV, 60 mm × 112 mm × 172 mm; FA, 80°; TR, 1,000 ms; TE, 35 ms. We used the University of Minnesota's CMRR-MB pulse sequence for SMS-EPI acquisition.

### Anatomical analysis

Whole-brain T1-weighted anatomical data were processed through the “recon-all” pipeline provided by the FreeSurfer neuroimaging analysis software (Fischl, 2012). The output was a model of the cortical surface that allowed for surface-based registration between functional and structural MRI data, ensuring that pRF and pSFT data were accurately mapped to the 3D space defined by the functional MRI volumes.

### Functional magnetic resonance imaging data preprocessing

All functional BOLD time series data were corrected for EPI distortions with a reverse phase-encoded method via the functional MRI of the Brain Software Library (FMRIB; Andersson et al., 2003). All fMRI fieldmap-corrected data were preprocessed with FreeSurfer Functional Analysis Stream (FS-FAST; Fischl, 2012), which applied standard motion correction procedures, Siemens slice timing correction, and boundary-based registration between functional and anatomical 3D spaces (Greve and Fischl, 2009). To allow for voxel-wise analysis of the data, no volumetric spatial smoothening was applied (FWHM = 0). Moreover, accurate volumetric alignment of functional data between scan runs was attained by applying robust rigid registration (Reuter et al., 2010). The target volume for alignment was designated as the middle time point of the first run from each session, while the middle time point of subsequent runs was used as the moveable volume for alignment.

### Population receptive field mapping

Every participant completed an independent population receptive field (pRF) mapping session. The pRF analysis was used to manually create ROI labels for early visual areas V1, V2, and V3. Each session involved 3–5 scans of both (A) rotating wedge stimuli and (B) bar sweep and expanding/contracting ring stimuli. All stimuli were presented on a mean luminance background and consisted of colored objects and faces of varying sizes over a pink noise background. During the stimulus presentation, participants performed a color change detection task at fixation, pressing a button when the fixation dot changed from red to white or white to red. The data acquired from these scans were analyzed with the *analyzePRF* toolbox for MATLAB (Kay et al., 2013), which estimates the visual field eccentricity, polar angle, and receptive field size for every voxel within the cortical ribbon of the occipital lobe.

### Population spatial frequency tuning mapping

**Figure 1.**
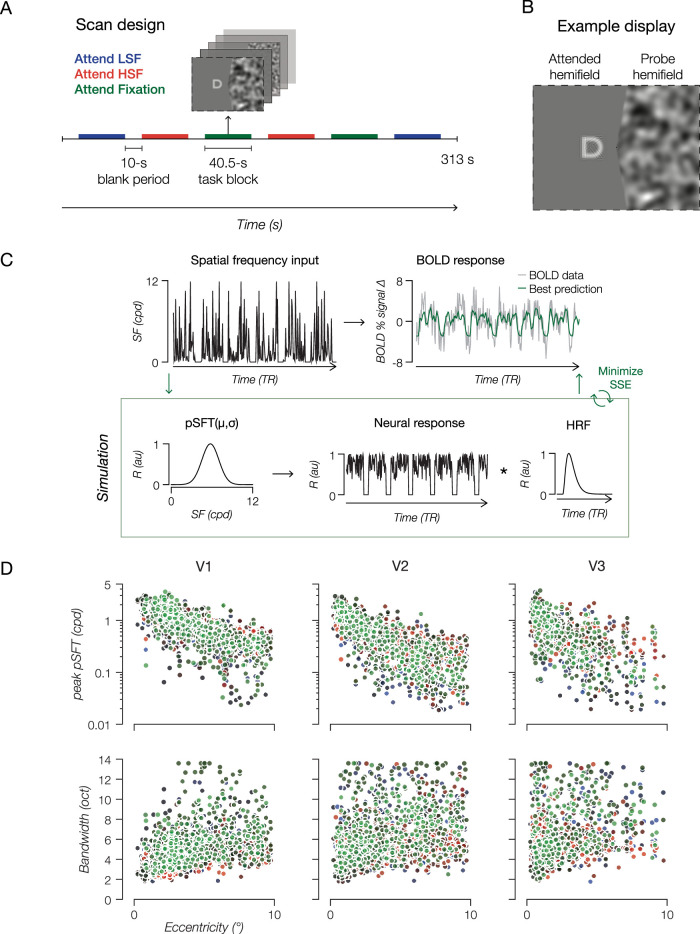
Experimental design and the pSFT model. ***A***, Within each scan (9 in total), participants completed two blocks of each condition in a pseudorandom order. ***B***, The visual display was split into an attended hemifield (task-relevant) and a probe hemifield (task-irrelevant). ***C***, For each voxel, condition-specific measured BOLD response was compared with a synthesized BOLD response (green trace) produced by the best prediction of pSFT parameters. ***D***, Estimated pSFT peak (top row) and bandwidth (bottom row) follow expected trends in every condition: decreased pSFT peak and increased bandwidth with pRF eccentricity and visual area. Each point represents a voxel that survived selection criteria. Voxels from all subjects are presented. Each subject has a unique shading in the scatter plots. Green represents estimates from the “Attend Fixation” condition, red from the “Attend HSF” condition, and blue from the “Attend LSF” condition. V1, *n* = 802; V2, *n* = 735; V3, *n* = 398. See also Extended Data Figure S1*A* and Extended Data Table S1.

Estimating population spatial frequency tuning (pSFT) from fMRI BOLD signals was contingent on the assumption that the BOLD signal is a product of a linear system (Boynton et al., 1996), an assumption often made in generating population receptive fields with fMRI (Dumoulin and Wandell, 2008). Additionally, due to the 10 s blank period in between blocks, we could concatenate voxel time series across every scan with respect to condition (“Attend LSF,” “Attend HSF,” or “Attend Fixation”), resulting in 18 spliced time series blocks per condition. Altogether, three sets of SF input and measured BOLD time series were fed into the pSFT model fitting pipeline (Fig. 1*C*).

We assumed that BOLD responses to spatial frequencies, R(f), can be characterized by a log-Gaussian distribution (Aghajari et al., 2020; Eq. 1), expressed as follows:

$$R(\left[\;f(t)\right])=\;e^{-\frac{{\left[\log\;(\;f(t))\;-\;\log(\mu )\right]}^{2}}{2\;\sigma^{2}}},\;(1)$$

where f(t) is the SF presented at time t, μ is the SF that produces the maximum response of the population (the “pSFT peak”), and σ is the linear SF tuning bandwidth— μ and σ being unknown. Because SF mapping stimuli were not presented during the blank periods in between blocks, the SF input during the blank periods was set to 0.0001 to avoid taking the log-transform of 0.

The population response, R[f(t)], was then convolved with a hemodynamic impulse response function, h(t), to generate a predicted BOLD signal, B(t):

$$B(t)=\;\beta_{0}+\beta \;\cdot R[\;f(t)]\;*\;h(t),\;(2)$$

where $\beta_{0}$ and β are unknown and represent the baseline and a scaling coefficient for the BOLD percent signal change, respectively. The hemodynamic impulse response function (HIRF), h(t), was a gamma function of the form:

$$h(t)=\;\frac{{\left(t/\tau \right)}^{\left(n-1\right)}e^{-(t/\tau )}}{\tau (n-1)!},\;(3)$$

where τ is the time constant (fixed to a value of 1.08), n is the phase delay (fixed to a value of 3), and t the delay between stimulus onset and the BOLD response (fixed to a value of 2.05; Boynton et al., 1996). We performed nonlinear regression to search for the most optimal μ, σ, β, and $\beta_{0}$ values, with the objective of minimizing the sum-of-squares error (SSE) between the predicted and measured BOLD percent signal change (via the *fmincon* function in MATLAB). Based on parameter bounds used in Aghajari et al. (2020), we constrained μ to values between 0.009 and 6, σ to values between 0.1 and 4, $\beta_{0}$ to values between −10 and 10, and β to values between −25 and 25.

To identify optimal starting values for nonlinear regression, we performed a preliminary coarse-to-fine grid search of model parameters. With the parameter constraints defined above, the coarse-grid search contained a combination of 10 logarithmically spaced μ values and 10 linearly spaced σ values, while β and $\beta_{0}$ were fixed to 1 and 0, respectively. The combination of values that produced the lowest SSE in the coarse-grid search were then used to generate a range of μ and σ values for the fine-grid search. Specifically, in the fine-grid search, 100 logarithmically spaced values for μ and 100 linearly spaced points for σ were generated between half and double the optimal coarse-grid search result for each parameter. β and $\beta_{0}$ were again fixed to 1 and 0, respectively. The combination of values that produced the lowest SSE in the fine-grid search was then used as the initial μ, σ, β, and $\beta_{0}$ values for nonlinear regression. If multiple values produced the minimum SSE, their average was used.

### Voxel selection

In each ROI, which was defined by the results of the pRF mapping procedure, we selected voxels whose pRF eccentricity was within 0.16–9.8°, pRF sizes >0.1° in diameter, polar angle within the probe aperture (if on the left hemifield, 100° < θ < 260°; if on the right hemifield, 280° < θ < 80°), and pRF *R*^2^ >10%. All reported effects on pSFT are contingent on this independent pRF analysis and thus avoid circular data selection errors (Stoll et al., 2022). As an additional voxel criterion for analysis inclusion, we selected voxels with pSFT *R*^2^ >10%, μ estimates between 0.01 and 5 cpd, and linear SF tuning bandwidth σ between 0.10 and 4 cpd. Lastly, we excluded voxels whose μ or σ estimates were three standard deviations away from the mean in any ROI and attention condition. These criteria left 100 ± 18 (50) [mean ± SEM (SD)] voxels in V1 [20 ± 2 (6)% remaining], 92 ± 12 (35) in V2 [19 ± 1 (5)% remaining], and 50 ± 6 (16) voxels in V3 [14 ± 1 (4)% remaining], which is comparable to previous counts (Vo et al., 2017; Foster and Ling, 2022) given that pSFT estimates were acquired in only one visual hemisphere.

### Attentional modulation index

To quantify changes in pSFT with attention, we computed voxel-wise attentional modulation indices (AMI) for pSFT peak and pSFT bandwidth. Calculating AMI involved taking the difference in parameter estimates between conditions and dividing this difference by their sum (Treue and Maunsell, 1996; Gandhi et al., 1999; Cohen and Maunsell, 2011; Van Es et al., 2018; Ni and Maunsell, 2019), followed by a percentage conversion:

$$\mathrm{AMI}\;(\text{\%})=\;\frac{A-B}{A+B}\;\times 100,\;(4)$$

### Dissimilarity from attended spatial frequency

To test for preference-dependent changes in pSFT with attention, we computed a voxel-wise dissimilarity metric between the baseline pSFT peak and the attended SF:

$$\mathrm{Dissimilarity}\;\mathrm{from}\;\mathrm{LSF}\;(\mathrm{octaves})=\log_{2}\;\left[\frac{\mu_{\mathrm{baseline}}}{0.5}\right],$$

$$\mathrm{Dissimilarity}\;\mathrm{from}\;\mathrm{HSF}\;(\mathrm{octaves})=\log_{2}\;\left[\frac{\mu_{\mathrm{baseline}}}{2.0}\right],$$

For example, voxels an octave above the attended LSF have a pSFT peak of 1 cpd in this space, while those same voxels would be an octave below the attended HSF.

### Line fitting

To test whether attentional modulation of pSFT is preference-dependent, we calculated subject-wise linear slopes between dissimilarity from the attended SF versus pSFT AMI, pRF eccentricity versus pSFT AMI, and pRF size versus pSFT AMI. The MATLAB function *polyfit* was used to find coefficients of a first-degree polynomial, fitting AMI best in a least-squares sense across voxels within an ROI and condition for each subject.

### Statistical analyses

All statistical analyses were performed in MATLAB. Subject averages were assumed to be normally distributed. To test for differences in task performance, pupil size, gaze position, gaze stability, and pSFT estimates between conditions, one-way ANOVA was performed at the group level. One-sample *t* tests and paired-sample *t* tests were performed on values that were computed relative to the baseline condition (e.g., attentional modulation indices and slopes from the “Attend LSF” and “Attend HSF” conditions). Effect sizes (Cohen's *d*) were calculated using the one- and paired-sample(s) formula to compare AMI within and between “Attend LSF” and “Attend HSF” conditions. To estimate 95% confidence intervals, we used bootstrap resampling (1,000 iterations), where data were sampled with replacement and Cohen's *d* was recomputed every iteration. The 2.5 and 97.5% percentiles of the bootstrapped distribution defined the confidence bounds. With *n* = 8, *α* = 0.05, and 80% power, we can reliably detect an effect size of ±1.16. Repeated measures correlation coefficients and *p*-values were calculated to test the relationship between change in peak and change in bandwidth (Bakdash and Marusich, 2017). Single, double, and triple asterisks correspond to Bonferroni-corrected *p*-values of 0.05, 0.01, and 0.001 to account for the possibility of Type I error in one-sample *t* tests and paired-sample *t* tests.

### Code accessibility

Code used for the experiment and data analyses can be found on the Open Science Framework at https://osf.io/meq9n/?view_only=e3b6a5af43514e2795d6243468db8a4c.

## Results

In an MRI scanner, participants performed one of three tasks in a blocked design (Fig. 1*A*): a low SF letter detection task at 0.5 cycles per degree (cpd; “Attend LSF” condition), a high SF letter detection task at 2 cpd (“Attend HSF” condition), and a luminance change detection task at fixation (“Attend Fixation” condition). The “Attend LSF” and “Attend HSF” conditions involved covertly attending to one of two superimposed letter streams (Fig. 1*B*, left hemifield) and reporting the detection of target letters J and K with a left and right button press, respectively. This spatial colocalization of the letters was designed to induce sufficient competition between the low and high SF letter streams, with the assumption that attending to a specific letter stream would promote attention to a specific band of SF content (Lee et al., 1999; White et al., 2015). Because SFs are processed by distinct subpopulations (Blakemore and Campbell, 1969; Ware and Mitchell, 1974), we hypothesized that selective attention to a low and high SF target distinct subpopulations across the visual field (Maunsell and Treue, 2006; Fang and Liu, 2019; Liu, 2019). These subpopulations are then read as a shift in peak SF and/or change in bandwidth when responses are measured across them (Ling et al., 2015).

To test this hypothesis, we measured and compared condition-specific voxel-wise pSFT in the task-irrelevant hemifield, where participants concurrently and passively viewed a range of bandlimited noise stimuli (Fig. 1*B*, right hemifield). This design exploits the well-established finding that the modulatory effects of feature-based attention “spread across the visual field” (Saenz et al., 2002), allowing us to interrogate changes in SF processing in a spatially unattended hemifield. Moreover, display statistics were identical in every block and separated by a 10 s blank period, allowing us to concatenate BOLD time series data with respect to a condition (Fig. 1*C*). Before evaluating the influence of attention on pSFT, we confirmed that in every condition pSFT peak decreased and pSFT bandwidth increased with pRF eccentricity and visual area (Fig. 1*D*; Sasaki et al., 2001; Henriksson et al., 2008; Aghajari et al., 2020; Broderick et al., 2022). Additionally, because the covertly attended location was identical between “Attend LSF” and “Attend HSF” conditions, and no significant differences in task performance, pupil size, gaze position, or gaze stability were found between conditions at the group level (Extended Data Fig. S1*B,C*), we could attribute any differences in pSFT between the “Attend LSF” and “Attend HSF” conditions to feature-based attention.

### Attentional modulation of pSFT

**Figure 2.**
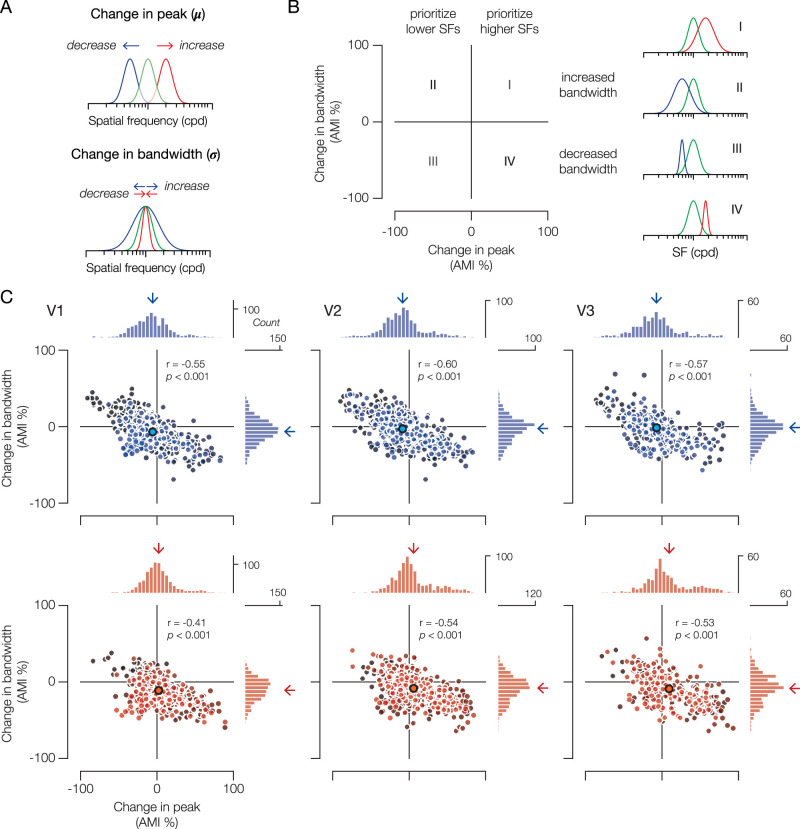
Attentional modulation of pSFT. ***A***, The key predictions afforded by the pSFT model are an increase/decrease in the pSFT peak and/or pSFT bandwidth. To facilitate the interpretation of attentional modulation of pSFT, the legend in ***B*** demonstrates the hypothesized modulatory strategies when the change in peak and bandwidth are plotted against one another. To the right are caricatures of each strategy (Quadrants I–IV). In ***C***, each dot represents a voxel's change in peak (*x*-axis) and bandwidth (*y*-axis) in the “Attend LSF” condition (blue, top row) and in the “Attend HSF” condition (red, bottom row). Each subject has a unique shading in the scatter plots. Histograms appended to the north and east walls of each plot reveal the distribution of AMI across all voxels in an ROI. Vertical and horizontal arrows above the histograms represent the group mean (*n* = 8) for change in peak and bandwidth, respectively. A dot at the intersection of these arrows is included for visibility. The repeated measures correlation coefficient and *p*-value are reported in the top right of each scatter plot. At the group level, the change in peak significantly differed between conditions in every ROI (V1 and V2 *p*s < 0.05; V3 *p* < 0.01). V1, *n* = 802; V2, *n* = 735; V3, *n* = 398. See also Table 1.

To quantify changes in pSFT due to attention, we computed voxel-wise attentional modulation indices (AMI) for pSFT peak and bandwidth. Calculating AMI involves taking the difference in parameter estimates between conditions, dividing the difference by their sum, and then converting the value to a percentage. By normalizing the difference in pSFT estimates between conditions by their total magnitude, this AMI measure emphasizes relative changes (Treue and Maunsell, 1996; Gandhi et al., 1999; Cohen and Maunsell, 2011; Van Es et al., 2018; Ni and Maunsell, 2019). Here, changes in peak and bandwidth due to attending the LSF or HSF were calculated relative to the “Attend Fixation” condition, which served as our baseline (Fig. 2*A*). If attentional modulation of pSFT in the task-irrelevant hemifield is evident and dependent on the attended SF, then we expected that the change in peak and/or bandwidth would significantly differ between “Attend LSF” and “Attend HSF” conditions.

Indeed, we found significant differences in the change in peak between “Attend LSF” and “Attend HSF” conditions at the group level [V1 and V2 *p*s < 0.05, V3 *p* < 0.01; Cohen's *d*: V1 = −1.29 (−3.64, −0.65), V2 = −1.29 (−2.47, −0.92), V3 = −1.80 (−6.35, −1.17); Table 1]. Generally, attending the LSF decreased the pSFT peak [Cohen's *d*: V1 = −0.38 (−1.89, 0.28), V2 = −0.73 (−2.40, −0.27), V3 = −0.53 (−1.31, 0.13)], while attending the HSF increased the pSFT peak [Cohen's *d*: V1 = 0.20 (−0.93, 0.80), V2 = 0.41 (−0.27, 1.26), V3 = 0.51 (−0.12, 1.68)]. In tandem, attending the LSF decreased the range of SFs that elicited a response [i.e., sharper tuning bandwidth; Cohen's *d*: V1 = −0.64 (−1.73, 0.03), V2 = −0.28 (−1.09, 0.46), V3 = −0.12 (−0.82, 0.87)] and attending the HSF even more so [Cohen's *d*: V1 = −1.05 (−2.25, −0.52), V2 = −0.95 (−2.44, −0.39), V3 = −0.72 (−1.72, −0.17)]. However, there were no significant differences in the change in bandwidth between conditions [*p*s > 0.05; Cohen's *d*: V1 = 0.97 (0.40, 2.37), V2 = 0.93 (0.32, 2.43), V3 = 0.82 (0.42, 1.78); Table 1]. Lastly, in V1, we found a significant decrease in BOLD amplitude in both “Attend LSF” and “Attend HSF” conditions (*p*s < 0.05), in agreement with the expected effects of covert spatial attention on the BOLD response of unattended locations (Gouws et al., 2014). We also found an increase in baseline in V1 in the “Attend HSF” condition (*p* < 0.05; Extended Data Table S1). Altogether, this suggests that feature-based attention to SF elicited global attractive shifts in preferred SF and increased selectivity.

**Table 1.** Group-level results for change in peak and bandwidth (AMI %)

Attend LSF Attend HSF Attend LSF versus Attend HSF						
	Change in peak			Change in bandwidth		
ROI	Mean ± 1 SEM	*P*	95% CI	Mean ± 1 SEM	*p*	95% CI
V1	−5.65 ± 5.22	0.944	−17.99, 6.69	−6.45 ± 3.54	0.334	−14.83, 1.92
	2.28 ± 4.13	1.000	−7.50, 12.05	−10.83 ± 3.64	0.062	−19.45, −2.22
	—	0.024*	−13.05, −2.80	—	0.087	0.60, 8.16
V2	−8.58 ± 4.16	0.234	−18.41, 1.25	−2.44 ± 3.07	1.000	−9.71, 4.82
	5.76 ± 4.97	0.853	−5.99, 17.51	−7.96 ± 2.96	0.093	−14.95, −0.97
	—	0.025*	−23.67, −5.02	—	0.101	0.56, 10.47
V3	−7.21 ± 4.86	0.544	−18.70, 4.28	−1.21 ± 3.56	1.000	−18.30, 1.31
	9.54 ± 6.65	0.583	−6.17, 25.26	−8.50 ± 4.15	0.239	−17.31, 1.20
	—	0.004**	−24.54, −8.96	—	0.162	−0.17, 14.73

Bonferroni-corrected *p*-values.

* *p* < 0.05.

** *p* < 0.01.

Interestingly, upon closer inspection, we found a range of modulation strategies in every visual area.

To elaborate, a change in pSFT for an individual voxel can fall under four strategies that consist of combinations of whether lower or higher SFs are prioritized (change in peak) and whether bandwidth is broadened or sharpened (change in bandwidth; Fig. 2*B*). When correlated against one another, what is the dominant relationship between changes in peak SF and bandwidth? In every visual area, we found a moderate negative correlation between attentional modulation of peak and bandwidth in both “Attend LSF” and “Attend HSF” conditions (*p*s < 0.001). In other words, a decrease in peak SF was associated with an increase in bandwidth, while an increase in peak SF was associated with a decrease in bandwidth (Fig. 2*C*).

Because the observed changes in pSFT were consistent across the early visual cortex, we wondered whether changes in tuning with feature-based attention to SF were driven by a feature-similarity mechanism (Maunsell and Treue, 2006; Liu, 2019), wherein subpopulations most similarly tuned to the attended feature experience increased response gain (Treue and Martínez-Trujillo, 1999; Martinez-Trujillo and Treue, 2004; Maunsell and Treue, 2006; Ling et al., 2009; Fang and Liu, 2019). We reasoned that if attentional modulation of pSFT were dependent on the dissimilarity between the baseline peak SF and the attended SF, then the rate of change in pSFT as a function of dissimilarity should significantly differ from zero.

To test for similarity-dependent modulation of pSFT, we first computed a voxel-wise dissimilarity metric between the baseline pSFT peak and attended SF (dissimilarityfromtheattendedSF $\left(\mathrm{octaves})=\log_{2}[\mu_{\mathrm{baseline}}/\mathrm{attended}\;\mathrm{SF}]\right)$. We then assessed subject-wise linear slopes between the baseline dissimilarity and change in peak and bandwidth in every visual area and condition. The sign of the slope—positive or negative—would indicate a repulsive or attractive effect for changes in peak SF, respectively. Similarly, for changes in bandwidth, positive or negative slopes would correspond to sharper-to-broader or broader-to-sharper bandwidths with increasing dissimilarity.

**Figure 3.**
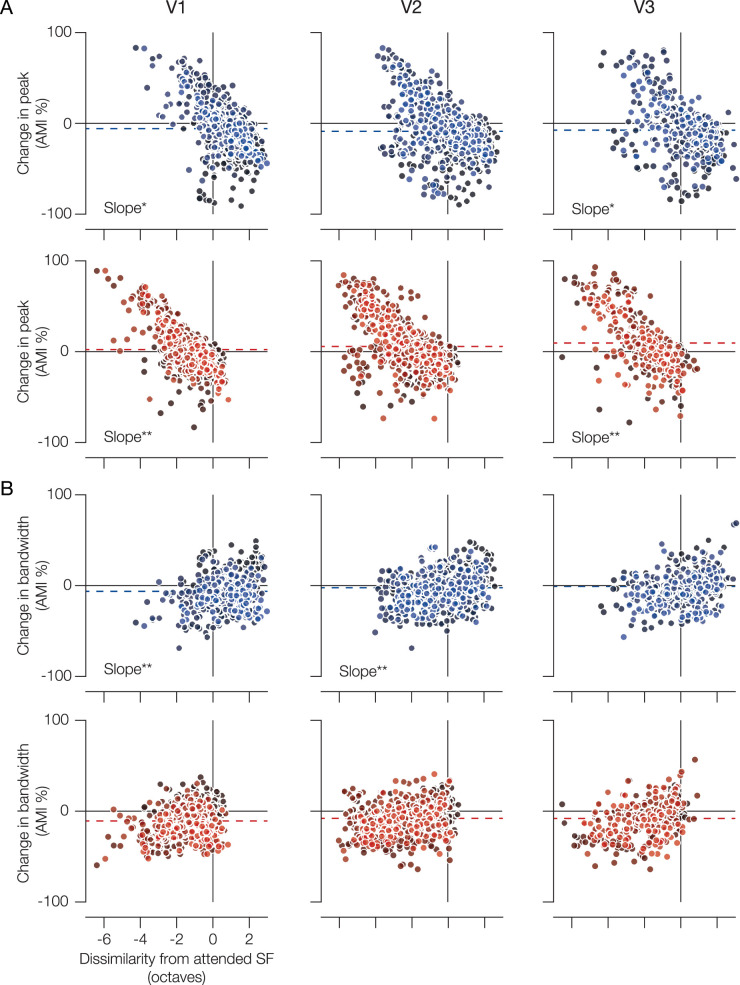
Attentional modulation of pSFT is preference-dependent. Voxel-wise changes in peak (***A***) and bandwidth (***B***) are reported as a percentage and as a function of the octave distance (i.e., dissimilarity) between the voxel's baseline pSFT peak and the attended SF (blue, LSF; red, HSF). Each subject has a unique shading in the scatter plots. Dashed lines represent the group mean (*n* = 8). Bonferroni-corrected *p < 0.05 and **p < 0.01. V1, *n* = 802; V2, *n* = 735; V3, *n* = 398. See also Figure 4 and Table 2.

**Figure 4.**
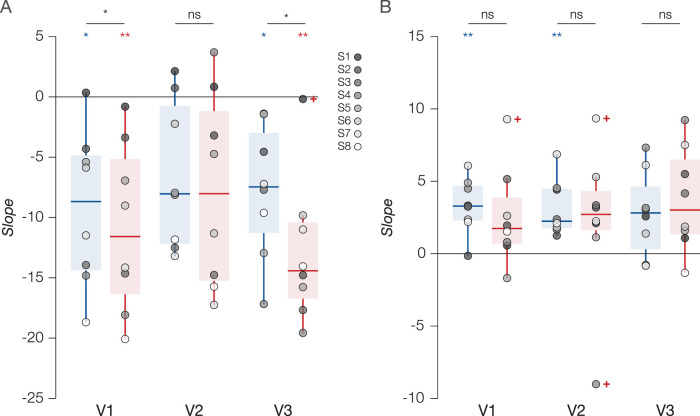
Attentional modulation of pSFT is dependent on the attended SF. ***A***, Box plot for baseline dissimilarity versus change in peak slopes. ***B***, Box plot for baseline dissimilarity versus change in bandwidth slopes. Each box depicts, from bottom to top, the minimum, 25th percentile, median, 75th percentile, and maximum slope across participants. Blue represents values from the “Attend LSF” condition, while red represents values from the “Attend HSF” condition. The “+” symbol represents outliers >1.5 times the interquartile range. Statistical test results reported above each plot were performed at the group level within conditions (blue/red) and between conditions (black). Bonferroni-corrected *p*-values. ^ns^*p* > 0.05, *p < 0.05, **p < 0.01. See also Table 2.

From V1 to V3, we found negative slopes for the change in peak as a function of baseline dissimilarity (Figs. 3*A*, 4*A*). More specifically, feature-based attention appeared to cause attractive shifts in peak SF when attention was directed to the lower SF (V1 and V3 *p*s < 0.05) and even stronger shifts when directed to the higher SF (V1 and V3 *p*s < 0.01). In V1 and V3, we found significant differences in the magnitude of attractive shifts between “Attend LSF” and “Attend HSF” conditions (*p*s < 0.05). While visual inspection suggests otherwise, we found no significant attractive shifts in peak SF in V2 within conditions nor between them (Fig. 4*A* and Table 2). In tandem, there was a positive relationship between baseline dissimilarity and change in bandwidth (Figs. 3*B*, 4*B*). This “sharper-to-broader” relationship across the preference space was significant only in V1 and V2 when attending the LSF (*p*s < 0.01; Table 1).

**Table 2.** Group-level results for linear slopes between dissimilarity from attended SF and change in peak and bandwidth (AMI/oct)

Attend LSF Attend HSF Attend LSF versus Attend HSF						
	Change in peak			Change in bandwidth		
ROI	Mean ± 1 SEM	*P*	95% CI	Mean ± 1 SEM	*p*	95% CI
V1	−9.27 ± 2.27	0.014*	−14.65, −3.89	3.31 ± 0.68	0.005**	1.71, 4.91
	−10.89 ± 2.46	0.009**	−16.70, −5.08	2.53 ± 1.18	0.211	−0.27, 5.33
	—	0.041*	0.44, 2.79	—	1.000	−2.02, 3.58
V2	−6.62 ± 2.15	0.053	−11.71, −1.54	3.13 ± 0.69	0.008**	1.50, 4.76
	−7.81 ± 2.84	0.085	−14.51, −1.10	2.21 ± 1.84	0.810	−2.15, 6.56
	—	1.000	−4.05, 6.43	—	1.000	−2.91, 4.76
V3	−7.76 ± 1.94	0.016*	−12.34, −3.17	2.75 ± 1.03	0.097	0.31, 5.19
	−12.86 ± 2.14	0.002**	−17.91, −7.81	3.70 ± 1.26	0.065	0.73, 6.67
	—	0.028*	1.71, 8.5	—	0.784	−2.79, 0.89

Bonferroni-corrected *p*-values.

* *p* < 0.05.

** *p* < 0.01.

### Attentional modulation of pSFT as a function of pRF

The spatial resolution hypothesis posits that attention resolves differences in spatial sampling (i.e., resolution) between foveal and peripheral populations (Yeshurun and Carrasco, 1998; Anton-Erxleben and Carrasco, 2013; Barbot and Carrasco, 2017). Although we could not measure changes in pRF with our experimental design, nor changes in pSFT in the attended hemifield, we wondered if there was a relationship between the baseline pRF (eccentricity and size; Extended Data Fig. S3) and changes in pSFT (Extended Data Text S1), as SF preferences vary systematically with eccentricity and correlate with size (Henriksson et al., 2008; Yu et al., 2010; Aghajari et al., 2020; Broderick et al., 2022; Kirsch and Kunde, 2023). Only in V3 and the “Attend HSF” condition did we find a significantly positive linear relationship between pRF eccentricity and change in peak (*p* < 0.01; Extended Data Fig. S2*A*, Extended Data Table S2). Additionally, we found a significantly positive relationship between pRF size and change in peak in both conditions in V3 (“Attend LSF,” *p* < 0.05; “Attend HSF,” *p* < 0.001; Extended Data Fig. S2*B*, Extended Data Table S3). Lastly, we found significant differences in the rate of change in pSFT peak as a function of pRF size and eccentricity “between” conditions in V3 (*p*s < 0.05), altogether supporting the possibility that selective attention to the LSF and HSF triggered unique changes in spatial resolution.

## Discussion

The existence of SF selective neurons in the early human visual system provides a window into how spatial patterns across the visual field are processed (Braddick, 1981). SF processing in the human visual cortex can be characterized as a set of parallel but interconnected narrow-band “channels” that together enable the perception of a wide range of spatial details across the retinal image (Wilson and Wilkinson, 1997; Kauffmann et al., 2014). If we assume that the convergence of neural populations across the early visual system are the constituents of these channels, then attention to SF should steer the center and/or width of SF-tuned responses toward the attended SF across the visuocortical hierarchy, as proposed in past research but not yet confirmed in humans (Schyns, 1998; Sowden and Schyns, 2006; David et al., 2008). Here, we addressed this gap in knowledge by acquiring pSFT in the early visual cortex (V1–V3) while manipulating selective attention to SF. We discovered profound shifts in tuning preferences for voxels in the early visual cortex, toward the SF content of an attended item. Below, we contextualize our findings and explore potential explanations for the observed changes in pSFT with attention.

In our task, participants were instructed to detect target letters in one of two superimposed letter streams that were low- and high-pass filtered, respectively. In both the “Attend LSF” and “Attend HSF” conditions, one could argue that texture segmentation at the covertly attended location is required to separate the relevant from irrelevant SF content (Yeshurun and Carrasco, 2000). Relative to a baseline condition where the letters were ignored, we found that feature-based attention triggered significantly distinct attractive shifts in SF preference, toward the attended SF and across early visual field maps of the unattended hemifield, along with increased selectivity for the newly preferred SF. From V1 to V3, LSF-preferring populations experienced the greatest increase in pSFT peak and decrease in bandwidth, while more HSF-preferring populations experienced the greatest decrease in pSFT peak and increase in bandwidth (Figs. 3, 4). Together, this demonstrates that changes in pSFT with attention are likely dependent on both the native SF tuning being sampled and the attended SF.

More specifically, we believe our findings are the consequence of covert spatial attention and feature-based attention selectively targeting neural subpopulations across the visual field (Saenz et al., 2002; White et al., 2015) to trigger attractive shifts along the task-relevant feature domain (Saenz et al., 2002; Womelsdorf et al., 2006; David et al., 2008; Klein et al., 2014; Liu, 2019; Chapman et al., 2023). While the shifts in preferred SF were significantly different between “Attend LSF” and “Attend HSF” conditions, the magnitude of the shifts within conditions was not (though this could be due to insufficient power). This is expected, in part because the SF tuning of a neural population sampled within a cortical column is ultimately constrained by its underlying neural architecture and thus cannot completely change its tuning profile (Blakemore and Campbell, 1969; Salinas and Abbott, 2001; David et al., 2008).

When moving out toward the periphery, the proportion of neurons within a population that is more selective for HSF decreases (Sasaki et al., 2001; Aghajari et al., 2020; Broderick et al., 2022). This relationship generates testable predictions for how pSFT might change with feature-based attention. In general, feature-based attention is theorized to operate via a feature-similarity gain mechanism, selectively targeting subpopulations tuned to the attended feature (Maunsell and Treue, 2006). Indeed, human psychophysical data from a 2-IFC task suggests that the feature-similarity gain model with asymmetrical surround suppression can characterize the effects of selective attention to SF (Fang and Liu, 2019). In other words, when an observer attends to an SF, feature-based attention should shift the peak SF response across a neural population toward the attended SF. For example, attending an LSF should target neurons tuned to that SF across the visual field. In central vision, LSF-preferring neurons would experience a gain in response, while HSF-preferring neurons are suppressed, together creating a shift toward lower SFs across a population (Ling et al., 2015). In peripheral vision, SF preferences are much lower relative to central vision, so this effect would instead create an observable shift toward higher SFs. While we believe feature-similarity gain is the primary mechanism, we did find a multitude of modulation strategies (Fig. 2*C*), which might be indicative of a mixture of attentional gain and preferential shifts in tuning at the single unit level (David et al., 2008; Ester et al., 2020).

Because SF tuning is tightly correlated with spatial receptive field characteristics (Altan et al., 2025), and both “Attend LSF” and “Attend HSF” conditions required covert spatial attention, our results likely align with the spatial resolution hypothesis, which proposes that covert attention resolves differences in spatial resolution between foveal and peripheral populations (Yeshurun and Carrasco, 1998; Anton-Erxleben and Carrasco, 2013; Barbot and Carrasco, 2017). There are signatures in our results that intersect with the predictions of the spatial resolution hypothesis. First, psychophysical studies that support the spatial resolution hypothesis reason that covert attention improves perception by selectively targeting small, HSF-selective RFs in the periphery (increasing resolution) and LSF-selective RFs in central vision (decreasing resolution; Yeshurun and Carrasco, 2000; Carrasco et al., 2006). In other words, attention can flexibly adjust spatial resolution with respect to task demands and native spatial sampling characteristics (Yeshurun and Carrasco, 1998; Yeshurun et al., 2008; Flevaris et al., 2014; Barbot and Carrasco, 2017; Van Es et al., 2018). Indeed, the rate of change in pSFT peak, as a function of the dissimilarity between the baseline pSFT peak and the attended SF, was more negative in the “Attend HSF” condition than in the “Attend LSF” condition in our study, perhaps because the HSF condition requires higher spatial sampling characteristics (i.e., higher pSFT peak and sharper tuning bandwidths) to resolve the high SF letters (Van Es et al., 2018). We also speculate that the observed changes in pSFT here should generalize when the visual system engages in global versus local processing, like in the case of Navon-type stimuli (Flevaris et al., 2014).

The flexibility of SF processing might be dependent on the observer's ability to diagnose the most relevant SF (Schyns, 1998); therefore, future studies might investigate the influence of perceptual learning and adaptation on pSFT (Fiorentini and Berardi, 1980; Sowden et al., 2002; Carrasco et al., 2006; Sowden and Schyns, 2006; Altan et al., 2025). The temporal dynamics of attentional modulation of pSFT might be another fruitful avenue of research, as the processing of LSFs and HSFs have unique temporal dynamics (Bredfeldt and Ringach, 2002; Mazer et al., 2002; Purushothaman et al., 2014). There is also a gap in knowledge for whether the covertly attended location and competing SFs at the attended location influence the magnitude of attractive shifts across the visual field (Majaj et al., 2002; Verghese et al., 2012), as physiological evidence has shown that attentional modulation is eccentricity-dependent (Roberts et al., 2007). Future work might leverage this to determine whether selective attention to low/high SF broadens/sharpens pRFs in an eccentricity-dependent manner (Altan et al., 2025). Addressing these questions will further adjudicate the malleability of SF processing with selective attention to SF.

In conclusion, we investigated the impact of selective attention to SF on pSFT in the early visual cortex. We found that feature-based attention to a low and high SF triggered unique attractive shifts in pSFT in the unattended hemifield, toward the attended SF. Our results support a dynamic human visual system, with spatial frequency, one of the building blocks of vision, bending to the will of attention.

## References

[B1] Aghajari S, Vinke LN, Ling S (2020) Population spatial frequency tuning in human early visual cortex. J Neurophysiol 123:773–785. 10.1152/jn.00291.2019 31940228 PMC7052645

[B2] Altan E, Morgan C, Dakin S, Schwarzkopf DS (2025) Spatial frequency adaptation modulates population receptive field sizes. eLife 13:RP100734. 10.7554/eLife.100734.1 PMC12470847 41002138

[B3] Andersson JLR, Skare S, Ashburner J (2003) How to correct susceptibility distortions in spin-echo echo-planar images: application to diffusion tensor imaging. Neuroimage 20:870–888. 10.1016/S1053-8119(03)00336-7 14568458

[B4] Anton-Erxleben K, Carrasco M (2013) Attentional enhancement of spatial resolution: linking behavioural and neurophysiological evidence. Nat Rev Neurosci 14:188–200. 10.1038/nrn3443 23422910 PMC3977878

[B5] Bakdash JZ, Marusich LR (2017) Repeated measures correlation. Front Psychol 8:456. 10.3389/fpsyg.2017.00456 28439244 PMC5383908

[B6] Barbot A, Carrasco M (2017) Attention modifies spatial resolution according to task demands. Psychol Sci 28:285–296. 10.1177/0956797616679634 28118103 PMC5555406

[B7] Blakemore C, Campbell FW (1969) On the existence of neurones in the human visual system selectively sensitive to the orientation and size of retinal images. J Physiol 203:237–260. 10.1113/jphysiol.1969.sp008862 5821879 PMC1351526

[B8] Boynton GM, Engel SA, Glover GH, Heeger DJ (1996) Linear systems analysis of functional magnetic resonance imaging in human V1. J Neurosci 16:4207–4221. 10.1523/JNEUROSCI.16-13-04207.1996 8753882 PMC6579007

[B9] Braddick O (1981) Spatial frequency analysis in vision. Nature 291:9–11. 10.1038/291009a0 7231529

[B10] Brainard DH (1997) The Psychophysics toolbox. Spat Vis 10:433–436. 10.1163/156856897X00357 9176952

[B11] Bredfeldt CE, Ringach DL (2002) Dynamics of spatial frequency tuning in macaque V1. J Neurosci 22:1976–1984. 10.1523/JNEUROSCI.22-05-01976.2002 11880528 PMC6758903

[B12] Broderick WF, Simoncelli EP, Winawer J (2022) Mapping spatial frequency preferences across human primary visual cortex. J Vis 22:3. 10.1167/jov.22.4.3 35266962 PMC8934567

[B13] Carandini M, Demb JB, Mante V, Tolhurst DJ, Dan Y, Olshausen BA, Gallant JL, Rust NC (2005) Do we know what the early visual system does? J Neurosci 25:10577–10597. 10.1523/JNEUROSCI.3726-05.2005 16291931 PMC6725861

[B14] Carrasco M, Loula F, Ho Y-X (2006) How attention enhances spatial resolution: evidence from selective adaptation to spatial frequency. Percept Psychophys 68:1004–1012. 10.3758/BF03193361 17153194

[B15] Carrasco M (2011) Visual attention: the past 25 years. Vision Res 51:1484–1525. 10.1016/j.visres.2011.04.012 21549742 PMC3390154

[B16] Chapman AF, Chunharas C, Störmer VS (2023) Feature-based attention warps the perception of visual features. Sci Rep 13:6487. 10.1038/s41598-023-33488-2 37081047 PMC10119379

[B17] Cohen MR, Maunsell JHR (2011) Using neuronal populations to study the mechanisms underlying spatial and feature attention. Neuron 70:1192–1204. 10.1016/j.neuron.2011.04.029 21689604 PMC3579499

[B18] Crossland MD, Sims M, Galbraith RF, Rubin GS (2004) Evaluation of a new quantitative technique to assess the number and extent of preferred retinal loci in macular disease. Vision Res 44:1537–1546. 10.1016/j.visres.2004.01.006 15126063

[B19] David SV, Hayden BY, Mazer JA, Gallant JL (2008) Attention to stimulus features shifts spectral tuning of V4 neurons during natural vision. Neuron 59:509–521. 10.1016/j.neuron.2008.07.001 18701075 PMC2948549

[B20] De Valois RL, Albrecht DG, Thorell LG (1982) Spatial frequency selectivity of cells in macaque visual cortex. Vision Res 22:545–559. 10.1016/0042-6989(82)90113-4 7112954

[B21] Dumoulin SO, Wandell BA (2008) Population receptive field estimates in human visual cortex. Neuroimage 39:647–660. 10.1016/j.neuroimage.2007.09.034 17977024 PMC3073038

[B22] Dux PE, Marois R (2009) The attentional blink: a review of data and theory. Atten Percept Psychophys 71:1683–1700. 10.3758/APP.71.8.1683 19933555 PMC2915904

[B23] Ester EF, Sprague TC, Serences JT (2020) Categorical biases in human occipitoparietal cortex. J Neurosci 40:917–931. 10.1523/JNEUROSCI.2700-19.2019 31862856 PMC6975303

[B24] Fang MWH, Liu T (2019) The profile of attentional modulation to visual features. J Vis 19:13. 10.1167/19.13.13 31747691 PMC6871543

[B25] Fiorentini A, Berardi N (1980) Perceptual learning specific for orientation and spatial frequency. Nature 287:43–44. 10.1038/287043a0 7412873

[B26] Fischl B (2012) Freesurfer. Neuroimage 62:774–781. 10.1016/j.neuroimage.2012.01.021 22248573 PMC3685476

[B27] Flevaris AV, Martínez A, Hillyard SA (2014) Attending to global versus local stimulus features modulates neural processing of low versus high spatial frequencies: an analysis with event-related brain potentials. Front Psychol 5:277. 10.3389/fpsyg.2014.00277 24782792 PMC3988377

[B28] Foster JJ, Ling S (2022) Feature-based attention multiplicatively scales the fMRI-BOLD contrast-response function. J Neurosci 42:6894–6906. 10.1523/JNEUROSCI.0513-22.2022 35868860 PMC9464014

[B29] Gandhi SP, Heeger DJ, Boynton GM (1999) Spatial attention affects brain activity in human primary visual cortex. Proc Natl Acad Sci U S A 96:3314–3319. 10.1073/pnas.96.6.3314 10077681 PMC15939

[B30] Gouws AD, Alvarez I, Watson DM, Uesaki M, Rogers J, Morland AB (2014) On the role of suppression in spatial attention: evidence from negative BOLD in human subcortical and cortical structures. J Neurosci 34:10347–10360. 10.1523/JNEUROSCI.0164-14.2014 25080595 PMC6608280

[B31] Greve DN, Fischl B (2009) Accurate and robust brain image alignment using boundary-based registration. Neuroimage 48:63–72. 10.1016/j.neuroimage.2009.06.060 19573611 PMC2733527

[B32] Henriksson L, Nurminen L, Hyvarinen A, Vanni S (2008) Spatial frequency tuning in human retinotopic visual areas. J Vis 8:5. 10.1167/8.10.5 19146347

[B33] Herrmann K, Heeger DJ, Carrasco M (2012) Feature-based attention enhances performance by increasing response gain. Vision Res 74:10–20. 10.1016/j.visres.2012.04.016 22580017 PMC3427403

[B34] Jazayeri M, Movshon JA (2006) Optimal representation of sensory information by neural populations. Nat Neurosci 9:690–696. 10.1038/nn1691 16617339

[B35] Kauffmann L, Ramanoël S, Peyrin C (2014) The neural bases of spatial frequency processing during scene perception. Front Integr Neurosci 8:37. 10.3389/fnint.2014.00037 24847226 PMC4019851

[B36] Kay K, Winawer J, Mezer A, Wandell BA (2013) Compressive spatial summation in human visual cortex. J Neurophysiol 110:481–494. 10.1152/jn.00105.2013 23615546 PMC3727075

[B37] Kirsch W, Kunde W (2023) Human perception of spatial frequency varies with stimulus orientation and location in the visual field. Sci Rep 13:17656. 10.1038/s41598-023-44673-8 37848541 PMC10582250

[B38] Klein BP, Harvey BM, Dumoulin SO (2014) Attraction of position preference by spatial attention throughout human visual cortex. Neuron 84:227–237. 10.1016/j.neuron.2014.08.047 25242220

[B39] Lee DK, Itti L, Koch C, Braun J (1999) Attention activates winner-take-all competition among visual filters. Nat Neurosci 2:375–381. 10.1038/7286 10204546

[B40] Lennie P (2003) The cost of cortical computation. Curr Biol 13:493–497. 10.1016/S0960-9822(03)00135-0 12646132

[B41] Ling S, Liu T, Carrasco M (2009) How spatial and feature-based attention affect the gain and tuning of population responses. Vision Res 49:1194–1204. 10.1016/j.visres.2008.05.025 18590754 PMC2696585

[B42] Ling S, Jehee JFM, Pestilli F (2015) A review of the mechanisms by which attentional feedback shapes visual selectivity. Brain Struct Funct 220:1237–1250. 10.1007/s00429-014-0818-5 24990408 PMC4282837

[B43] Liu T (2019) Feature-based attention: effects and control. Curr Opin Psychol 29:187–192. 10.1016/j.copsyc.2019.03.013 31015180 PMC6756988

[B44] Majaj NJ, Pelli DG, Kurshan P, Palomares M (2002) The role of spatial frequency channels in letter identification. Vision Res 42:1165–1184. 10.1016/S0042-6989(02)00045-7 11997055

[B45] Martinez-Trujillo JC, Treue S (2004) Feature-based attention increases the selectivity of population responses in primate visual cortex. Curr Biol 14:744–751. 10.1016/j.cub.2004.04.028 15120065

[B46] Maunsell JHR (2015) Neuronal mechanisms of visual attention. Annu Rev Vis Sci 1:373–391. 10.1146/annurev-vision-082114-035431 28532368 PMC8279254

[B47] Maunsell JHR, Treue S (2006) Feature-based attention in visual cortex. Trends Neurosci 29:317–322. 10.1016/j.tins.2006.04.001 16697058

[B48] Mazer JA, Vinje WE, McDermott J, Schiller PH, Gallant JL (2002) Spatial frequency and orientation tuning dynamics in area V1. Proc Natl Acad Sci U S A 99:1645–1650. 10.1073/pnas.022638499 11818532 PMC122244

[B49] Moeller S, Yacoub E, Olman CA, Auerbach E, Strupp J, Harel N, Uğurbil K (2010) Multiband multislice GE-EPI at 7 tesla, with 16-fold acceleration using partial parallel imaging with application to high spatial and temporal whole-brain fMRI. Magn Reson Med 63:1144–1153. 10.1002/mrm.22361 20432285 PMC2906244

[B50] Ni AM, Maunsell JHR (2019) Neuronal effects of spatial and feature attention differ due to normalization. J Neurosci 39:5493–5505. 10.1523/JNEUROSCI.2106-18.2019 31068439 PMC6616284

[B51] Pelli DG, Robson JG, Wilkins AJ (1988) The design of a new letter chart for measuring contrast sensitivity. Clin Vis Sci 2:187–199. ISSN 0887-6169/88

[B52] Pestilli F, Carrasco M, Heeger DJ, Gardner JL (2011) Attentional enhancement via selection and pooling of early sensory responses in human visual cortex. Neuron 72:832–846. 10.1016/j.neuron.2011.09.025 22153378 PMC3264681

[B53] Pouget A, Dayan P, Zemel R (2000) Information processing with population codes. Nat Rev Neurosci 1:125–132. 10.1038/35039062 11252775

[B54] Purushothaman G, Chen X, Yampolsky D, Casagrande VA (2014) Neural mechanisms of coarse-to-fine discrimination in the visual cortex. J Neurophysiol 112:2822–2833. 10.1152/jn.00612.2013 25210162 PMC4254879

[B55] Reuter M, Rosas HD, Fischl B (2010) Highly accurate inverse consistent registration: a robust approach. Neuroimage 53:1181–1196. 10.1016/j.neuroimage.2010.07.020 20637289 PMC2946852

[B56] Roberts M, Delicato LS, Herrero J, Gieselmann MA, Thiele A (2007) Attention alters spatial integration in macaque V1 in an eccentricity-dependent manner. Nat Neurosci 10:1483–1491. 10.1038/nn1967 17906622 PMC2673551

[B57] Saenz M, Buracas GT, Boynton GM (2002) Global effects of feature-based attention in human visual cortex. Nat Neurosci 5:631–632. 10.1038/nn876 12068304

[B58] Salinas E, Abbott LF (2001) Coordinate transformations in the visual system: how to generate gain fields and what to compute with them. In: Progress in brain research (Nicolelis MAL, ed), pp 175–190. Amsterdam, Netherlands: Elsevier. 10.1016/s0079-6123(01)30012-2 11480274

[B59] Sasaki Y, Hadjikhani N, Fischl B, Liu AK, Marret S, Dale AM, Tootell RBH (2001) Local and global attention are mapped retinotopically in human occipital cortex. Proc Natl Acad Sci U S A 98:2077–2082. 10.1073/pnas.98.4.2077 11172078 PMC29384

[B60] Schyns PG (1998) Diagnostic recognition: task constraints, object information, and their interactions. Cognition 67:147–179. 10.1016/S0010-0277(98)00016-X 9735539

[B61] Serences J, Saproo S, Scolari M, Ho T, Muftuler L (2009) Estimating the influence of attention on population codes in human visual cortex using voxel-based tuning functions. Neuroimage 44:223–231. 10.1016/j.neuroimage.2008.07.043 18721888

[B62] Simoncelli EP, Olshausen BA (2001) Natural image statistics and neural representation. Annu Rev Neurosci 24:1193–1216. 10.1146/annurev.neuro.24.1.1193 11520932

[B64] Sowden PT, Schyns PG (2006) Channel surfing in the visual brain. Trends Cogn Sci 10:538–545. 10.1016/j.tics.2006.10.007 17071128

[B63] Sowden PT, Rose D, Davies IRL (2002) Perceptual learning of luminance contrast detection: specific for spatial frequency and retinal location but not orientation. Vision Res 42:1249–1258. 10.1016/S0042-6989(02)00019-6 12044757

[B65] Stoll S, Infanti E, de Haas B, Schwarzkopf DS (2022) Pitfalls in post hoc analyses of population receptive field data. Neuroimage 263:119557. 10.1016/j.neuroimage.2022.119557 35970472 PMC7617406

[B66] The MathWorks Inc. (2017) MATLAB version: 9.3.0.713579 (2017b).

[B67] Treue S, Martínez-Trujillo JC (1999) Feature-based attention influences motion processing gain in macaque visual cortex. Nature 399:575–579. 10.1038/21176 10376597

[B68] Treue S, Maunsell JHR (1996) Attentional modulation of visual motion processing in cortical areas MT and MST. Nature 382:539–541. 10.1038/382539a0 8700227

[B69] Van Der Kouwe AJW, Benner T, Salat DH, Fischl B (2008) Brain morphometry with multiecho MPRAGE. Neuroimage 40:559–569. 10.1016/j.neuroimage.2007.12.025 18242102 PMC2408694

[B70] Van Es DM, Theeuwes J, Knapen T (2018) Spatial sampling in human visual cortex is modulated by both spatial and feature-based attention. Elife 7:e36928. 10.7554/eLife.36928 30526848 PMC6286128

[B71] Verghese P, Kim Y-J, Wade AR (2012) Attention selects informative neural populations in human V1. J Neurosci 32:16379–16390. 10.1523/JNEUROSCI.1174-12.2012 23152620 PMC3543832

[B72] Vo VA, Sprague TC, Serences JT (2017) Spatial tuning shifts increase the discriminability and fidelity of population codes in visual cortex. J Neurosci 37:3386–3401. 10.1523/JNEUROSCI.3484-16.2017 28242794 PMC5373124

[B73] Ware C, Mitchell DE (1974) The spatial selectivity of the tilt aftereffect. Vision Res 14:735–737. 10.1016/0042-6989(74)90072-8 4424240

[B74] White AL, Rolfs M, Carrasco M (2015) Stimulus competition mediates the joint effects of spatial and feature-based attention. J Vis 15:7. 10.1167/15.14.7 26473316 PMC5077277

[B75] Wilson HR, Wilkinson F (1997) Evolving concepts of spatial channels in vision: from independence to nonlinear interactions. Perception 26:939–960. 10.1068/p260939 9509156

[B76] Womelsdorf T, Anton-Erxleben K, Pieper F, Treue S (2006) Dynamic shifts of visual receptive fields in cortical area MT by spatial attention. Nat Neurosci 9:1156–1160. 10.1038/nn1748 16906153

[B77] Wu W (2024) We know what attention is!. Trends Cogn Sci 28:304–318. 10.1016/j.tics.2023.11.007 38103983

[B78] Xu J, Moeller S, Auerbach EJ, Strupp J, Smith SM, Feinberg DA, Yacoub E, Uğurbil K (2013) Evaluation of slice accelerations using multiband echo planar imaging at 3T. Neuroimage 83:991–1001. 10.1016/j.neuroimage.2013.07.055 23899722 PMC3815955

[B80] Yeshurun Y, Carrasco M (1998) Attention improves or impairs visual performance by enhancing spatial resolution. Nature 396:72–75. 10.1038/23936 9817201 PMC3825508

[B81] Yeshurun Y, Carrasco M (2000) The locus of attentional effects in texture segmentation. Nat Neurosci 3:622–627. 10.1038/75804 10816320

[B79] Yeshurun Y, Montagna B, Carrasco M (2008) On the flexibility of sustained attention and its effects on a texture segmentation task. Vision Res 48:80–95. 10.1016/j.visres.2007.10.015 18076966 PMC2638123

[B82] Yu H-H, Verma R, Yang Y, Tibballs HA, Lui LL, Reser DH, Rosa MGP (2010) Spatial and temporal frequency tuning in striate cortex: functional uniformity and specializations related to receptive field eccentricity. Eur J Neurosci 31:1043–1062. 10.1111/j.1460-9568.2010.07118.x 20377618

[B83] Zhang W, Luck SJ (2009) Feature-based attention modulates feedforward visual processing. Nat Neurosci 12:24–25. 10.1038/nn.2223 19029890

